# Epidemiology of Adult Soft-Tissue Sarcomas in Germany

**DOI:** 10.1155/2018/5671926

**Published:** 2018-04-04

**Authors:** Catherine W. Saltus, Brian Calingaert, Sean Candrilli, Maria Lorenzo, Yulia D'yachkova, Thorsten Otto, Uwe Wagner, James A. Kaye

**Affiliations:** ^1^RTI Health Solutions, 307 Waverly Oaks Rd., Suite 101, Waltham, MA, USA; ^2^RTI Health Solutions, 200 Park Offices Drive, Research Triangle Park, NC, USA; ^3^Lilly Research Centre, Eli Lilly and Company Limited, Erl Wood Manor, Sunninghill Road, Windlesham, Surrey GU20 6PH, UK; ^4^Eli Lilly GmbH, Koelblgasse 6-8, Vienna 1030, Austria; ^5^Lilly Deutschland GmbH, Werner-Reimers-Straße 2-4, 61352 Bad Homburg, Germany

## Abstract

We conducted a retrospective cohort study using data compiled from the regional German cancer registries by the Centre for Cancer Registry Data (ZfKD) at the Robert Koch Institut (RKI) to describe the epidemiology of adult soft-tissue sarcomas (STS) in Germany in 2003–2012, focusing on advanced STS. We identified 33,803 incident adult cases of STS (other than the Kaposi sarcoma and gastrointestinal stromal tumors). The incidence of STS was 6.05 (95% confidence interval (CI), 5.82–6.29) per 100,000 in 2012 (4,079 cases). During 2003–2012, the most common histologic categories were leiomyosarcoma (19%), liposarcoma (16%), and STS not otherwise specified (14%). The overall STS-specific mortality rate in 2012 was 2.31 (95% CI, 2.06–2.57) per 100,000, and the median overall survival from initial diagnosis was 5.83 (95% CI, 5.50–6.08) years. Using STS mortality rates as a proxy for incidence of advanced STS in Germany and applying the age- and sex-specific rates to the corresponding German population, we estimated that 1,581 incident adult advanced STS cases occurred in Germany in 2012. Our findings contribute to a refined understanding of the population burden of STS in Germany, including the number of patients with advanced STS who may be candidates for systemic treatment.

## 1. Introduction

Soft-tissue sarcomas (STS) are a heterogeneous group of malignant neoplasms derived from cells of mesodermal origin that are widely distributed in the body within organs and in other sites designated as connective tissues. The World Health Organization (WHO) International Classification of Diseases for Oncology, Third Edition (ICD-O-3), identifies more than 50 histologic subtypes of STS based on inferred cell type of origin and other histologic and molecular features [1]. The WHO has subsequently revised its classification of sarcomas twice; the most recent classification was published in 2013 [2].

The epidemiology of adult STS is challenging to characterize. ICD-10 (International Statistical Classification of Diseases and Related Health Problems, 10th Revision) codes can be used to identify cases. However, ICD-10 codes for STS specify anatomic sites (connective tissues) rather than histology. This classification underestimates the true incidence and prevalence of STS. For example, in a comprehensive analysis of data from the Surveillance, Epidemiology, and End Results (SEER) Program of the United States (US) National Cancer Institute, Toro et al. [3] estimated that less than half (47.9%) of all STS cases arose from connective tissues; the remaining STS cases were identified in sites such as skin, uterus, retroperitoneum, stomach, and small intestine. Similarly, in a study conducted by the West Midlands Cancer Intelligence Unit (WMCIU) in the United Kingdom, only 41% of all STS cases arose from connective tissues; other STS cases arose from the female genital organs, skin, digestive organs, retroperitoneum or peritoneum, or other sites [4].

Cases of STS can be identified more comprehensively in cancer registries using ICD-O-3 codes than using ICD-10 codes. ICD-O-3 codes have three parts: (1) the topography code (anatomical site of origin, similar to ICD-10 codes), (2) the morphology code (specifying histology), and (3) the behavior code (e.g., malignant, in situ, benign, or uncertain). The morphology code allows identification of STS arising in organs and other anatomic sites in addition to those arising in connective tissues per se.

Recent data on the epidemiology of STS in Germany are sparse. Estimates of STS incidence have been reported for only one federal state [5], were derived from medical insurance claims data [5], used ICD-10 coding [5, 6], may not have reliably distinguished incident from prevalent cases [5], included pediatric age groups [6, 7] and/or all sarcomas [7], and did not report specifically on patients with advanced (i.e., metastatic or unresectable locally advanced) STS [5–7]. It is important to obtain a more accurate understanding of the epidemiology of adult STS and particularly of advanced disease. This will provide quantitative information useful for considering the value of new treatment for advanced adult STS. We used German cancer registry data to describe the population burden of adult STS (other than the Kaposi sarcoma and gastrointestinal stromal tumors (GIST)), that is, its incidence, prevalence, mortality, and the number of advanced adult STS cases estimated to occur annually. We used the data from the most recent decade available, 2003–2012, to provide stable estimates for several measures, and in addition, we focused on 2012 for some analyses in order to present the most current estimates possible.

## 2. Materials and Methods

This was a retrospective cohort study of adult patients diagnosed in Germany in 2003–2012. We used data compiled from the regional cancer registries in Germany by the Centre for Cancer Registry Data (ZfKD) at the Robert Koch Institut (RKI) and population census data obtained by the German Statistical Office. Due to completeness and data availability considerations, the analyses of incidence and prevalence used data from the nine federal states in Germany, and the analysis of cancer-specific mortality was based on data from four of these nine states.

### 2.1. Data Sources

The RKI maintains the ZfKD, which comprises data from regional population-based cancer registries operated within each of 16 German federal states. The ZfKD was established following the Federal Cancer Registry Data Act [8], which came into effect on August 2009, and requires all federal states within Germany to have a state-wide cancer registry that annually transfers data to the ZfKD. (Prior to the Federal Cancer Registry Data Act, the Federal Cancer Surveillance Unit, established at the RKI in 1983, served as a central site for evaluating regional cancer registry data, but participation was not mandatory and not all states contributed [9].) The data compiled by the ZfKD are available across all 16 German federal states. However, the start of registration and completeness with which new cancer cases are included vary across registries. Completeness is estimated by comparing each region's mortality-to-incidence ratio to that of reference regions. A detailed explanation of this process can be found in Section 2.1 of *Cancer in Germany 2011/2012* [6]. According to the current estimates, averaged across the years 2003 (or start of registry) through 2012, the nine federal states achieved an estimated completeness of at least 90% across all cancers combined and also specifically across sarcomas (identified by ICD-10 codes C40-C41 and C45–C49). Additional details about the completeness of individual registries can be found in Supplementary Table S1. Since identifying a high proportion of all cases that occur in the population is critical for estimating cancer incidence, we considered these nine states with at least 90% completeness of data to provide the most accurate data for estimating cancer incidence in Germany: Bavaria, Bremen, Hamburg, Lower Saxony, North Rhine-Westphalia, Rhineland-Palatinate, Saarland, Saxony, and Schleswig-Holstein. Four of these states (Bavaria, Bremen, Saarland, and Schleswig-Holstein) consistently reported cause of death to the ZfKD and were used to calculate cancer-specific mortality rates.

Anonymized data were extracted by the RKI for the 10 most recent years of available information at the time of the data request (January 1, 2003, through December 31, 2012) according to specifications described in Study Population and were provided to RTI Health Solutions to conduct the analyses presented herein.

Annual population estimates during the study period, stratified by age and sex for each of the 16 federal states, were obtained from the German Federal Statistical Office, and they represent the population on December 31 of each year. These data were used as denominators in calculating incidence and mortality rates in the states from which data were used and for extrapolating estimates from the studied states to the overall German population.

### 2.2. Study Population

We included cases with a first diagnosis of STS (other than the Kaposi sarcoma and GIST) between January 1, 2003, and December 31, 2012. STS cases were identified by ICD-O-3 morphology and topography codes (Supplementary Tables S2 and S3) with a behavior code “3” (malignant). Instances of more than one STS tumor diagnosis for the same individual were rare; for these, only the first diagnosis with a qualifying STS code was assessed to determine if the case met eligibility criteria. Cases were excluded if they were aged younger than 18 years at the initial diagnosis of STS or if the individual at any time had a diagnosis of the Kaposi sarcoma, GIST, or bone sarcoma (Supplementary Table S4). Although cases for this study were identified using ICD-O-3 codes, they are also associated with ICD-10 diagnosis codes in the ZfKD data.

Cases whose STS was documented by death certificate only (“DCO cases”) were included in initial counts of the study population but were excluded from all other analyses because the date of diagnosis, which is missing for some of these cases, is needed for incidence and survival analyses.

### 2.3. Analysis

All analyses were descriptive and exploratory in nature; no hypotheses were tested. Epidemiologic measures and 95% confidence intervals were estimated. Data assessed at cohort entry included demographics and tumor characteristics (histologic subtype, anatomical location, stage, and grade).

Histologic subtypes of sarcomas were identified using ICD-O-3 codes and grouped into broader categories based on the WHO Classification of Tumors of Soft Tissue and Bone [2] (see Supplementary Table S3):

- Adipocytic tumors (liposarcomas)
- Fibroblastic/myofibroblastic tumors (which include fibrosarcomas and other histologic subtypes)
- So-called fibrohistiocytic tumors (hereafter, “fibrohistiocytic tumors,” which include sarcomas designated by the older term “malignant fibrohistiocytic sarcoma” (MFH) and other subtypes)
- Smooth muscle tumors (leiomyosarcomas)
- Pericytic (perivascular) tumors (pericytic sarcomas)
- Skeletal muscle tumors (rhabdomyosarcomas)
- Vascular tumors of soft tissue (angiosarcomas)
- Nerve sheath tumors
- Tumors of uncertain differentiation
- Undifferentiated/unclassified sarcomas (include sarcomas designated by the newer term “undifferentiated pleomorphic sarcoma” (UPS) and other subtypes)

Anatomic locations of primary tumors were also grouped into broader categories (see Supplementary Table S3).

Using data from the nine federal states with at least 90% data completeness, we extrapolated age- and sex-specific incidence rates to the entire German population to estimate the overall incidence of adult STS in Germany. We also estimated incidence rates separately for the five most commonly occurring histologic STS categories. All incidence rates are reported per 100,000 inhabitants. Age- and sex-specific 5-year partial prevalence counts of adult STS on December 31, 2012, were calculated using data from the nine federal states with at least 90% completeness. (Five-year partial prevalence counts include individuals alive on that date who were diagnosed with STS in the previous 5 years.)

These counts were then extrapolated to the full German population by dividing them by the corresponding age- and sex-specific population totals among the nine states and then multiplying these results by the full German population for that age and sex category. The age- and sex-specific counts were then summed across all categories to create an overall 5-year STS partial prevalence estimate for Germany.

We estimated survival for cases in the nine states with at least 90% completeness. We excluded 24 cases with missing year of death from survival analyses (less than 0.3% of those who died). Overall survival from the date of STS diagnosis was estimated using the Kaplan–Meier method. Results are presented overall and stratified by sex, age, and histologic category (for the five most common). The Hall and Wellner method was used to derive 95% confidence bands for survival estimates. These confidence bands assure that the probability is 95% that each of the individual confidence interval estimates simultaneously covers its respective true survival probability [10].

Causes of death are reported in ZfKD as ICD-10 codes. We calculated the number of deaths attributable to cancer (C00–C97 and D00-D48) among the study cohort in 2012, stratified by age and sex, in the four states (Bavaria, Bremen, Saarland, and Schleswig-Holstein) that consistently reported the cause of death to the ZfKD. We used all cancer-attributed causes of death in this analysis because restricting to those coded C46–C49, which do not apply to approximately half of STS cases, would have resulted in substantial underestimation of the number of STS-related deaths in the study cohort. Cancer-specific mortality rates were estimated per 100,000 inhabitants.

We estimated the number of new cases of advanced STS (either diagnosed with or progressed to advanced STS) in 2012 using cancer-specific mortality as a proxy. The assumptions underlying this method are (1) all patients with advanced STS die from the disease, (2) patients with a diagnosis of STS who subsequently die of cancer had advanced STS at death, and (3) both the incidence of advanced STS and the mortality rate related to advanced STS are stable over time. Age- and sex-specific cancer-specific mortality rates were used in this analysis. Note that this method does not imply that all patients who developed advanced STS died of the disease in the same year they developed advanced STS. Under steady-state conditions, when the incidence of advanced STS and the mortality rate for advanced STS are constant, this method provides an estimate of the annual number of incident advanced STS cases, conditional on the assumptions listed above. To the extent that some cancer-related deaths among patients with STS are due to an unrelated malignancy, this method can overestimate the number of STS-related deaths and the number of new cases of advanced STS in 2012.

All analyses were conducted using SAS version 9.4 (SAS Institute, Inc., 2011; Cary, North Carolina).

## 3. Results

### 3.1. Patient Characteristics

Altogether, 36,265 cases of adult STS were identified by the tumor registries in the 16 federal states of Germany during 2003–2012 (Table 1). Three of these registries joined after 2003 and therefore did not report cases for the entire 10-year period. The great majority of cases (86%) were confirmed based on tumor histology; however, 7% were documented by death certificate only (DCO cases). For reasons stated previously, DCO cases were excluded from this study, leaving 33,803 cases. These were evenly distributed between males and females; the median age was 65 years, and 26% of cases were aged 75 years or older (Table 1).

Overall, the most common histologic categories were leiomyosarcoma (19%), liposarcoma (16%), sarcoma not otherwise specified (NOS) (14%), fibroblastic/myofibroblastic (12%), fibrohistiocytic (10%), and tumors of uncertain differentiation (9%) (Table 1). The order was fairly consistent across all state registries. The most common anatomic locations of primary tumor were lower extremity (20%), trunk (15%), head or neck (12%), miscellaneous (11%), and upper extremity (8%). This pattern was consistent across states with the exception of Hamburg, which was more likely to code sites as miscellaneous (28%). Among the 33,803 non-DCO cases, 20,609 (61.0%) had an ICD-10 diagnosis code C46–C49 (not shown in Table 1).

Information on stage at diagnosis (Union for International Cancer Control Stage Grouping) was missing for 27,646 cases (81.8%) (not shown in Table 1). Stages I, II, III, and IV were reported for 2,081 (6.2%), 833 (2.5%), 1,024 (3.0%), and 2,219 (6.6%) cases, respectively. Tumor grade at diagnosis was missing for 15,167 cases (44.9%) (not shown in Table 1). Grades 1, 2, and 3 were reported for 4,267 (12.6%), 5,038 (14.9%), and 9,331 cases (27.6%), respectively. Stage and grade were not used for subsequent analyses because of the high proportions of missing data.

### 3.2. Incidence and Prevalence

During the study period, 24,777 non-DCO cases were identified by the nine state registries with 90% or greater data completeness. Incidence rates for 2003–2012 and for 2012 only are presented in Table 2. For the entire study period, the incidence rates range from 5.75 per 100,000 in Hamburg to 8.24 in Saarland. The total incidence rate for 2012 (6.05 per 100,000) is close to that for the entire study period (6.10 per 100,000).

Incidence rates stratified by sex and age for the year 2012 are reported in Table 3. Overall STS rates increased substantially and consistently with increasing age. For individuals aged 65–74 years, the rates are somewhat higher for males than females, and for those aged 75 years or older, the rate in males is almost twice the rate in females. For both sexes combined, the rates range from 2.06 cases per 100,000 for those aged 18–44 years to 15.75 for those aged 75 years or older. Applying the age- and sex-specific incidence rates in Table 3 to the corresponding strata of the entire German population yields an estimated total of 4,079 incident cases of STS in 2012. Incidence rates for the five most common histologic categories of adult soft-tissue sarcoma are shown in Table 4.

The estimated 5-year partial prevalence count of STS among adults in Germany on December 31, 2012, was 14,554 individuals. Prevalence estimates were approximately equal for males and females, and prevalence estimates were also similar among those aged 18–64 years and those aged 65 years and older (data not shown).

### 3.3. Survival and Mortality Rates

Survival estimates were based on 24,753 STS cases at risk; among these, 10,382 died of any cause during the 10-year study period. The numbers of deaths stratified by age, sex, and histologic category are shown in Table 5, with corresponding numbers of patients at risk of dying, 5-year survival estimates, and median survival times.

**Figure 1:**
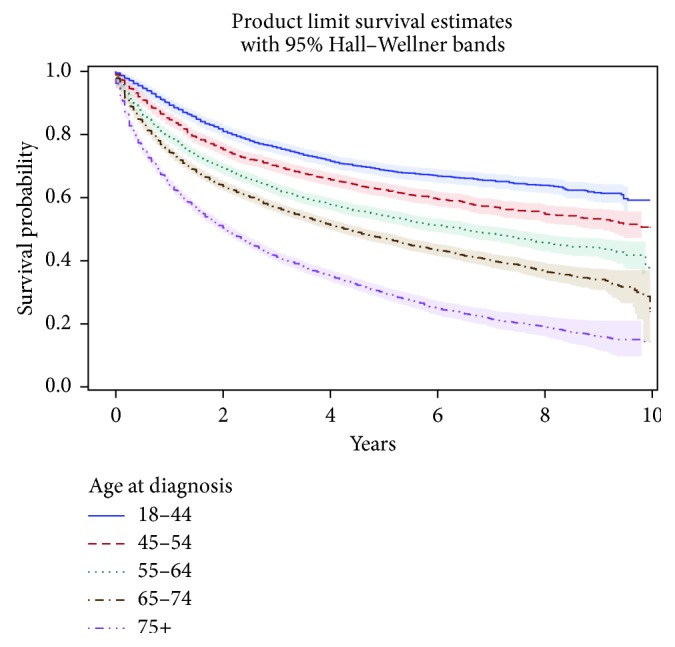
Estimated survival (with 95% CIs) of patients with soft-tissue sarcoma, by age, among cases diagnosed from 2003 to 2012 in nine German states (*n* = 24,753).

**Figure 2:**
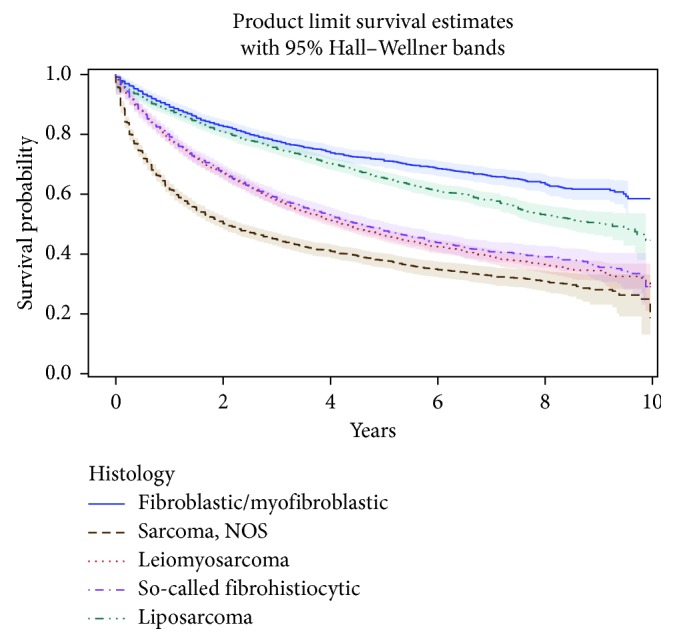
Estimated survival (with 95% CIs) of patients with soft-tissue sarcoma, by histologic category, among cases diagnosed from 2003 to 2012 in nine German states (*n* = 17,604).

Median overall survival from initial STS diagnosis was 5.83 years (95% CI, 5.50–6.08). The 1-year overall survival probability was 0.77 (95% CI, 0.77-0.78) (not shown in Table 5), and the 5-year probability was 0.52 (95% CI, 0.52–0.53). Survival was similar for males and females. However, a progressive decline in survival with increasing age is apparent in stratified analysis (Table 5 and Figure 1). Five-year survival estimates stratified by histologic category range from 0.41 (95% CI, 0.39–0.43) for sarcoma NOS to 0.73 (95% CI, 0.71–0.75) for fibroblastic/myofibroblastic tumors (Table 5 and Figure 2).

In the four state registries with adequate cause-of-death information, 2,898 cancer-specific deaths were reported among cases diagnosed during the 10-year study period. Over the first 5 years of the study period, the number of cancer-specific deaths increased each year. This occurred because cases were selected based on the year of sarcoma diagnosis, and during the early years of follow-up, the observation time available to assess survival experience was limited. Consequently, mortality counts in the later years of the study are expected to yield a more valid estimate of the current total number of annual deaths related to STS in Germany (and of the STS-related age- and sex-specific mortality rates) than mortality counts from earlier years. Stabilization of the cancer-specific death counts in the last 5 years of the study period is consistent with the known limited prognosis of patients with advanced STS, who are unlikely to survive longer than 5 years after their disease becomes advanced.

Of note for later discussion, among the 2,898 STS cases with a cancer-specific cause of death, 1,455 (50.2%) were attributed to an ICD-10 code C46–C49. However, this underestimates the proportion of deaths related to STS in the study population because, as had been expected (see Introduction) and as reported previously, only 61.0% of non-DCO cases in the study population were associated with an ICD-10 code C46–C49 at the time of diagnosis. Therefore, it is more informative to consider that among the 2,898 STS cases with a cancer-specific cause of death, 1,794 (61.9%) were associated with a code C46–C49 at diagnosis. Among these 1,794 cases, 1,325 (73.9%) had a code C46–C49 recorded as their cause of death.

The cancer-specific mortality rates among STS cases stratified by sex and age in 2012 are displayed in Table 6. Similar to results of all-cause mortality, cancer-specific mortality rates increased with increasing age, and the total rates were similar between males and females (2.28 versus 2.33 per 100,000).

### 3.4. Incidence of Advanced Soft-Tissue Sarcoma

Cancer-specific mortality rates in 2012 (stratified by age and sex) were applied to the entire German population to serve as a proxy for the annual incidence of advanced STS. Using this method, we estimate that 1,581 new cases of advanced STS were diagnosed in patients aged 18 years or older in 2012 in Germany (Table 7).

## 4. Discussion

This study describes the recent epidemiology of adult STS in Germany and the annual incidence of advanced STS. During the 10-year study period, 2003–2012, we estimated the incidence rate to be 6.10 per 100,000 overall; in 2012, we estimated it to be 6.05 per 100,000. Incidence varied by histologic category and increased markedly with age. Median overall survival from initial STS diagnosis was 5.83 years and was worse for older patients and for those with sarcoma histology that was not otherwise specified.

Although population-based cancer registry data are well suited for investigating incidence and survival related to adult STS in general, the occurrence of advanced STS can be determined directly in such data only for patients who presented initially with advanced disease. A patient's physician may know that a patient whose STS initially diagnosed at an earlier stage later experienced progression to an advanced extent of disease, but such information on cancer progression is not routinely reported to registries. Therefore, we used the cancer-specific mortality rate among patients with STS as a proxy for the incidence of advanced STS.

Among the assumptions required for this method to be valid, there is good evidence that advanced STS frequently results in death (clinical trial survival estimates with longer follow-up show <10% of patients with advanced STS alive after 10 years) [11]. Moreover, consistent with another of the assumptions, the age-standardized incidence and mortality rates for malignant soft-tissue tumors in Germany have been almost constant since 1999 [6]. However, approximately one-quarter of cases in the present study who had cancer-specific death among those with an ICD-10 STS code at diagnosis may have actually died of an unrelated malignancy. We assume that a similar proportion of cases not associated with a code C46–C49 at diagnosis also may have died of an unrelated malignancy. Therefore, the method we used results in considerable overestimation of the STS-related deaths in the study population. However, restriction of the analysis to cases associated with a code C46–C49 would have resulted in substantial underestimation of the number of STS-related deaths and the number of new cases of advanced STS in 2012 because these codes do not identify a large proportion of the STS cases included in the study. Deaths are not attributed to specific ICD-O-3 histology codes in cancer registry data, so it was not possible to use the same classification for cancer-specific causes of death as was used for case identification.

Only one other recently published study reported population-based epidemiological data on STS in Germany. Trautmann et al. [5] conducted a retrospective cohort study using data from AOK (Allgemeine Ortskrankenkasse), a statutory health insurance agency covering approximately 60% of all inhabitants of the federal state of Saxony. The age-standardized (European standard population) incidence of STS between 2007 and 2011 ranged from 4.3 to 6.1 per 100,000 (average annual incidence was 4.5 per 100,000; 95% CI, 2.6–7.9 per 100,000). This study differs from ours in that patients with STS were identified using only ICD-10 code C49. This approach underestimates the complete incidence of STS because it does not capture cases with primary tumors in organs or other sites that are not considered “connective tissues.” In addition, standardization of results to the European population would also tend to produce lower incidence estimates for the German population since the German population (median age: 45.8 years) is somewhat older, on average, than the overall European population (median age: 42.6 years) [12]. Diagnoses were not confirmed by independent medical record review in that study but rather only through attestation in the insurance records by physicians. In the present study, most diagnoses were confirmed by histology, although not additionally by an independent pathologist. Moreover, the case definition used in the Trautmann et al. [5] study may not have reliably distinguished incident from prevalent cases, while cancer registry cases are recorded at the time of initial diagnosis (apart from DCO cases, which we excluded from analysis). Our study adds to information about the German federal state of Saxony published by Trautmann et al. [5] in that we have reported case counts, incidence, and survival for an additional eight federal states and for a more extensive population (not limited to those insured by AOK).

A more recently published study of pooled data from the German Centre for Cancer Registry Data for registries with “sufficient completeness” estimated the age-adjusted (European standard) incidence of all sarcomas (including GIST and bone sarcomas) [7]. In 2014, the age-adjusted incidence of sarcomas per 100,000 was 6.9 for males and 6.5 for females of all ages in Germany. Soft-tissue sarcomas comprised 90% of the cases. Therefore, it can be calculated that the age-adjusted incidence of soft-tissue sarcomas estimated from this study, still including GIST, would be 6.2 and 5.9 per 100,000 for males and females, respectively. Since Ressing et al. [7] included individuals of all ages in their study population and since the incidence of STS in pediatric age groups is lower than that in the adult population, we would expect that study to have produced a slightly lower incidence estimate than we observed. Despite these differences, the results reported by Ressing et al. [7] are fairly comparable to our findings. However, Ressing et al. [7] did not report any information on the incidence of *advanced* STS in Germany, which our study does provide.

In the report “Cancer in Germany, 2011/2012,” [6] a joint publication of the RKI and ZfKD, several measures were estimated to describe the epidemiology of soft-tissue sarcomas in 2012 (Table 3.13 in the reference). The ICD-10 codes C46–C49 were used to identify cases, and those of all ages were included in the analysis. As noted by the authors of the RKI-ZfKD report, these codes fail to identify approximately 45% of soft-tissue sarcomas that are assigned to other organs in the ICD-10 system. Moreover, pediatric and adolescent cases were included in the RKI-ZfKD analyses as well as cases of the Kaposi sarcoma—none of which were included in the study reported here. Although the RKI-ZfKD results cannot therefore be expected to be identical to those in the present study, they are of similar magnitude. For example, comparing the RKI-ZfKD analyses to results from the present study (in parentheses) yielded a total of 3,510 (4,079) incident cases and 1,541 (1,581) deaths among patients with STS in 2012. The higher incidence rates in the present study (6.05 per 100,000) than found in the RKI-ZfKD analyses for either males (4.6 per 100,000) or females (4.2 per 100,000) may be related to extrapolation in the present study from the state registries with high completeness to the entire German population. Moreover, the additional adult STS cases identified in the present study through the use of ICD-O-3 codes are likely more numerous than the collective number of pediatric/adolescent and Kaposi sarcoma cases included in the RKI-ZfKD analyses.

Several population-based registry collaborations provide data on STS in Europe. The EUROCARE (Survival of Cancer Patients in Europe) project includes data on more than 21 million cancer diagnoses provided by 116 cancer registries in 30 European countries [13]. The RARECARE (Surveillance of Rare Cancers in Europe) project includes data from 89 national and regional population-based cancer registries [14] in 21 European countries [15]. Cancer Incidence in Five Continents (CI5) is the result of a long collaboration between the International Agency for Research on Cancer and the International Association of Cancer Registries and includes information from regional or national registries worldwide [16]. A study of patients with STS using EUROCARE, CI5, and United Nations population projections (1992–1996) found that the crude incidence rate of STS (including GIST) per 100,000 in the European Union among individuals aged 20 years and older was 2.0–4.7 in men and 1.9–4.1 in women [17]. Included in these estimates are fibrosarcoma, nerve sheath tumor, malignant fibrous histiocytoma, synovial, alveolar, leiomyosarcoma, liposarcoma, rhabdomyosarcoma, GIST, dermatofibrosarcoma, angiosarcoma, and unspecified STS. Using data from RARECARE, Gatta et al. [18] estimated that the crude incidence of STS in Europe between 1988 and 2002 was 4.74 per 100,000. Although these estimates include the GIST diagnoses, we expect crude incidence estimates for Germany to be higher than estimates for Europe because of the older age distribution in the German population relative to the overall European population and the marked increase in STS incidence with increasing age.

We are aware of only one population-based European study that reported incidence of STS by histologic type. A registry study using data from the Netherlands Cancer Registry (2006–2011) included 2,709 patients with STS (excluding the Kaposi sarcoma, GIST, uterine sarcoma, and sarcoma of the skin) and estimated that the overall annual age-standardized incidence rate was 2.7 per 100,000 population [19]. This age-standardized incidence rate is lower than the crude incidence rate (6.1 per 100,000 in 2003–2012) from the present study. The distribution of diagnoses among the most frequent histologic types was leiomyosarcoma (21%), sarcoma NOS (20%), liposarcoma (19%), fibrosarcoma (10%), and undifferentiated sarcoma (10%). Although the authors did not report the coding system used for capturing cases of STS, we expect that the overall age-standardized incidence rate would be lower in the Netherlands study than in ours due to the exclusion of uterine sarcoma in the former. Still, the relative distribution of histologic types in the Netherlands study is generally consistent with our findings.

A study conducted in the US using SEER data from 1978 to 2001 reported age-adjusted (US standard population) incidence (per 100,000) for leiomyosarcoma (1.23), malignant fibrous histiocytoma (0.88), sarcomas NOS (0.65), liposarcoma (0.59), and dermatofibrosarcoma (0.5) [3]. That study used the ICD-O-3 coding system to identify cases. Although the study by Gutierrez et al. [20] used data from an earlier time period than our study, incidence rates by histologic subtype from that study are also reasonably consistent with our findings.

We reviewed only two studies, one in the US [20] and one in the Netherlands [19] (discussed previously), provided mortality or survival estimates stratified by disease stage, and these were limited to information at the time of diagnosis. The US study was a registry study in 8,249 patients with STS (including GIST) using data from the Florida Cancer Data System from 1981 to 2004 [20]. Median overall survival for all patients with STS was 25 months, and 5- and 10-year overall survival estimates were 23.6% and 7.9%, respectively [20]. Sarcomas were identified using the ICD-O-3 classification. In our study, median overall survival for all patients with STS was 5.83 years (95% CI, 5.50–6.08), which is more than twice the estimate from Gutierrez et al. [20]. This may be related to improvements in diagnoses and treatment, resulting in longer survival times during the more recent years included in our study. The 5-year survival probability was also higher in our study than in that of Gutierrez et al. [20] (52% versus 24%, resp.).

The present study estimated epidemiologic measures of all STS (other than the Kaposi sarcoma and GIST) in Germany based on the national cancer registry data, which are a population-based source of information collected for public health purposes. Diagnoses of STS from cancer registry data using ICD-O-3 coding should be more complete than those from studies of medical records because cancer registry diagnoses are specified by histology and by anatomic location rather than only by anatomic location (ICD-10 codes). Furthermore, a substantial proportion of soft-tissue sarcomas arise within organs or other anatomic locations (e.g., uterus, retroperitoneum, and visceral organs) that are not soft tissues per se. We used information on the quality of data from contributing cancer registries (such as data completeness and the proportion of DCO cases) to improve the quality of the estimates provided in this report. Finally, by making a few simple assumptions that are reasonably consistent with the available data, we were able to estimate the incidence of *advanced* STS in Germany.

Despite these strengths, the present study has several additional limitations:

- The only follow-up information routinely available in cancer registry data is date of death and, for some registries, cause of death. Only 5 of the 16 state registries provided cause-of-death information, and for 1 of those 5, cause of death was missing for a significant portion of the deaths. Therefore, to mitigate this limitation, cancer-specific mortality rates and the estimate of annual occurrence of advanced STS were based on data from only 4 of 16 German states. This also required assuming that the age- and sex-specific rates estimated from these 4 states apply to all of Germany and that most cancer-related deaths in the study population were due to STS. The latter assumption is largely supported by our findings on ICD-10 STS codes.
- Identification of patients with STS was conducted using relevant ICD-O-3 codes; the advantage of this method was described earlier; however, as in any study using coded data, any coding errors that may have occurred could lead to some misclassification of patients.
- Undifferentiated pleomorphic sarcoma (UPS), one of the most common subtypes in the current WHO STS classification, did not occur frequently in this retrospective analysis of tumors diagnosed since 2003. This subtype was likely captured within the categories of fibrohistiocytic sarcoma, which included the former malignant fibrous histiocytoma, and undifferentiated/unclassified sarcomas.
- Other pragmatic limitations are inherent in using any cancer registry data for a study such as this. For example, completeness of reporting of incident cases and availability of clinical details varied among the states contributing to the RKI national data, and overall there was a high proportion of missing information on tumor grade and stage at diagnosis. To mitigate this in our study, the available data judged to have the highest quality were used to generate the incidence and mortality estimates presented.

## 5. Conclusions

Using cancer registry data in Germany, we identified 33,803 incident adult cases of STS (other than the Kaposi sarcoma and gastrointestinal stromal tumors, and excluding STS cases identified only by death certificate) based on ICD-O-3 codes specifying histology. The annual incidence of STS was stable over the study period, and the most common histologic categories were leiomyosarcoma, liposarcoma, and STS not otherwise specified. Both STS incidence and mortality rates increased with age. Using the STS mortality rate as a proxy for incidence of advanced STS in Germany and applying the age- and sex-specific rates to the corresponding German population, we estimated that 1,581 incident adult advanced STS cases occurred in Germany in 2012.

To our knowledge, this is the first study to provide comprehensive, German population–based epidemiological estimates of the incidence, mortality, and survival duration for adult patients with STS (other than the Kaposi sarcoma and GIST) and an estimate of the number of new cases of *advanced* STS occurring in Germany. To enhance the validity of our results, pathologically defined and highly specific diagnostic codes were used to identify the study population from state cancer registries, and only those state registries with the best available data quality were included in the analyses. Our findings contribute to an enhanced understanding of the population burden related to STS in the adult population in Germany and, in particular, the subpopulation with advanced STS who may be candidates for treatment with a systemic therapy.

## Figures and Tables

**Table 1:** Demographic and clinical characteristics of soft-tissue sarcoma cases from 2003 to 2012 in all 16 German states.

Characteristic	Number (%)
All cases including death certificate-only cases (*n*, %)	36,265 (100)
Type of diagnosis confirmation^a^ (*n*, %)	
Histology of primary tumor	31,037 (86)
Death certificate only	2,462 (7)
Others (including autopsy, clinical diagnostics, clinically without specified diagnostic, cytology, histology of metastasis, and specific tumor markers)	1,040 (3)
Missing	1,726 (5)

*Results below this row exclude death certificate-only cases*	
Cases excluding death certificate-only cases (study population) (*n*, %)	33,803 (100)
Sex (*n*, %)	
Female	16,924 (50)
Male	16,879 (50)
Age at diagnosis (years)	
Mean (SD)	62.7 (16.4)
Median	65
IQR (Q1, Q3)	51, 75
Distribution (*n*, %)	
18–44	5,141 (15)
45–54	5,028 (15)
55–64	6,008 (18)
65–74	8,770 (26)
75+	8,856 (26)
Year of initial diagnosis (*n*, %)	
2003–2005	7,775 (23)
2006–2008	10,301 (30)
2009–2011	11,924 (35)
2012	3,803 (11)
Histologic category (*n*, %)	
Leiomyosarcoma	6,501 (19)
Liposarcoma	5,242 (16)
Sarcoma, NOS	4,720 (14)
Fibroblastic/myofibroblastic tumors	4,072 (12)
Fibrohistiocytic tumors	3,263 (10)
Tumors of uncertain differentiation	3,108 (9)
Angiosarcoma	2,176 (6)
Undifferentiated/unclassified sarcomas	2,018 (6)
Nerve sheath tumors	1,202 (4)
Rhabdomyosarcoma	782 (2)
Malignant neoplasm arising in soft tissues, NOS	508 (2)
Pericytic tumors	211 (1)
Anatomic location of primary tumor (*n*, %)	
Lower extremity	6,687 (20)
Trunk	4,933 (15)
Head or neck	3,934 (12)
Miscellaneous	3,653 (11)
Upper extremity	2,811 (8)
Uterus	1,949 (6)
Retroperitoneal	1,921 (6)
Gastrointestinal	1,843 (5)
Pelvis (nonvisceral)	1,667 (5)
Breast	1,291 (4)
Genitourinary	1,188 (4)
Heart, mediastinum, lung, or pleura	1,169 (3)
Unknown	383 (1)
Gynecologic (other than uterus)	374 (1)

IQR: interquartile range; NOS: not otherwise specified; Q1: first quartile; Q3: third quartile; SD: standard deviation; ^a^categories are mutually exclusive and presented exactly as reported in the registry.

**Table 2:** All soft-tissue sarcoma cases, incidence rates per 100,000 inhabitants in nine German states, by registry.

Federal state registries with at least 90% completeness	2003–2012^a^		2012			
	Cases	IR	Cases	Population^b^	IR	95% CI
Bavaria	6,045	5.89	620	10,430,583	5.94	(5.49–6.43)
Bremen	365	6.55	33	555,794	5.94	(4.09–8.34)
Hamburg	850	5.75	70	1,460,601	4.79	(3.74–6.06)
Lower Saxony	3,806	5.87	361	6,452,746	5.60	(5.03–6.20)
North Rhine-Westphalia	7,061	6.02	926	14,617,533	6.34	(5.93–6.76)
Rhineland-Palatinate	2,252	6.78	182	3,340,484	5.45	(4.69–6.30)
Saarland	714	8.24	75	849,874	8.83	(6.94–11.06)
Saxony	2,277	6.28	263	3,484,777	7.55	(6.66–8.52)
Schleswig-Holstein	1,407	6.06	105	2,337,981	4.49	(3.67–5.44)
Total	24,777	6.10	2,635	43,530,373	6.05	(5.82–6.29)

CI: confidence interval; IR: incidence rate; ^a^2006–2012 for North Rhine-Westphalia since this state joined the registry in 2006; ^b^population comprises those aged 18 years or older on December 31, 2012; the year-specific population of each state was used for each year's rate calculation, but only 2012 population data are displayed in the table.

**Table 3:** Incidence rates of adult soft-tissue sarcoma per 100,000 inhabitants in 2012 in nine German states, stratified by age and sex.

Age (years)	Cases	Population	IR	95% CI
Male				
18–44	180	8,714,770	2.07	(0.18–2.39)
45–54	187	4,394,069	4.26	(0.37–4.91)
55–64	221	3,310,963	6.68	(0.58–7.62)
65–74	337	2,655,113	12.69	(1.14–14.12)
75+	429	1,971,614	21.76	(1.98–23.92)
Total	1,354	21,046,529	6.43	(0.61–6.79)
Female				
18–44	176	8,551,019	2.06	(0.18–2.39)
45–54	206	4,323,478	4.77	(0.41–5.46)
55–64	239	3,430,281	6.97	(0.61–7.91)
65–74	274	2,977,364	9.20	(0.82–10.36)
75+	386	3,201,702	12.06	(1.09–13.32)
Total	1,281	22,483,844	5.70	(0.54–6.02)
Both sexes				
18–44	356	17,265,789	2.06	(0.19–2.29)
45–54	393	8,717,547	4.51	(0.41–4.98)
55–64	460	6,741,244	6.82	(0.62–7.48)
65–74	611	5,632,477	10.85	(1.00–11.74)
75+	815	5,173,316	15.75	(1.47–16.87)
Total	2,635	43,530,373	6.05	(0.58–6.29)

CI: confidence interval; IR: incidence rate. Data in this table are limited to the nine federal states with at least 90% completeness.

**Table 4:** Incidence rates for the five most common histologic categories of adult soft-tissue sarcoma per 100,000 inhabitants in nine German states, 2012.

Histologic category	Cases	IR	95% CI
Leiomyosarcoma	485	1.11	(1.02–1.22)
Liposarcoma	422	0.97	(0.88–1.07)
Sarcoma, NOS	385	0.88	(0.80–0.98)
Fibroblastic/myofibroblastic	344	0.79	(0.71–0.88)
Fibrohistiocytic	194	0.45	(0.39–0.51)

CI: confidence interval; IR: incidence rate; NOS: not otherwise specified. Population used for the denominator is those aged 18 years and older on December 31, 2012 (43,530,373).

**Table 5:** Five-year survival and median survival, overall and by sex, age, and histologic category (for the five most common) among cases diagnosed from 2003 to 2012 in nine German states.

Group	Number at risk	Died		Five-year survival probability (95% CI)^a^	Median survival time in years (95% CI)^a^
		*n*	(%)		
Overall	24,753	10,382	(41)	0.52 (0.52–0.53)	5.83 (5.50–6.08)
Sex					
Male	12,367	5,220	(41)	0.52 (0.51–0.53)	5.67 (5.33–6.00)
Female	12,386	5,162	(41)	0.53 (0.52–0.54)	6.00 (5.59–6.58)
Age at diagnosis (years)					
18–44	3,810	933	(26)	0.71 (0.70–0.73)	NE
45–54	3,615	1,100	(31)	0.65 (0.63–0.67)	NE
55–64	4,357	1,646	(36)	0.58 (0.56–0.59)	8.33 (7.67–NE)
65–74	6,368	2,837	(46)	0.50 (0.48–0.51)	4.92 (4.58–5.33)
75+	6,603	3,866	(56)	0.33 (0.31–0.34)	2.25 (2.08–2.34)
Histologic category					
Fibroblastic/myofibroblastic	2,986	701	(26)	0.73 (0.71–0.75)	NE
Leiomyosarcoma	4,814	2,106	(46)	0.50 (0.48–0.52)	5.00 (4.59–5.58)
Liposarcoma	3,872	1,050	(26)	0.69 (0.67–0.71)	NE
Sarcoma, NOS	3,583	1,896	(51)	0.41 (0.39–0.43)	2.42 (2.17–2.83)
Fibrohistiocytic	2,349	1,086	(46)	0.49 (0.47–0.51)	4.92 (4.33–5.41)

CI: confidence interval; *n*: the number of cases who died; NE: not estimable; NOS: not otherwise specified; ^a^calculated using the Kaplan–Meier method. 24 cases with missing year of death were excluded from this analysis (representing less than 0.3% of the cases who died); data in this table are limited to the nine federal states with at least 90% completeness.

**Table 6:** Cancer-specific mortality rates in patients diagnosed with soft-tissue sarcoma per 100,000 inhabitants in four German states in 2012, by sex and age.

Age (years)	Deaths (*n*)	Population	Rate (95% CI)
Male			
18–44	15	2,888,628	0.52 (0.29–0.86)
45–54	18	1,435,925	1.25 (0.74–1.98)
55–64	23	1,058,370	2.17 (1.38–3.26)
65–74	48	872,392	5.50 (4.06–7.30)
75+	53	618,462	8.57 (6.42–11.21)
Total	157	6,873,777	2.28 (1.94–2.67)
Female			
18–44	13	2,836,651	0.46 (0.24–0.78)
45–54	18	1,414,017	1.27 (0.75–2.01)
55–64	38	1,096,789	3.47 (2.45–4.76)
65–74	37	961,609	3.85 (2.71–5.30)
75+	64	991,389	6.46 (4.97–8.24)
Total	170	7,300,455	2.33 (1.99–2.71)
Both sexes			
18–44	28	5,725,279	0.49 (0.33–0.71)
45–54	36	2,849,942	1.26 (0.89–1.75)
55–64	61	2,155,159	2.83 (2.17–3.64)
65–74	85	1,834,001	4.64 (3.70–5.73)
75+	117	1,609,851	7.27 (6.01–8.71)
Total	327	14,174,232	2.31 (2.06–2.57)

CI: confidence interval. Data in this table are based on the four federal states that consistently reported the cause of death, all four of which have at least 90% completeness of data; in Bremen, the seven deaths with missing cause of death were assumed to be cancer-specific deaths.

**Table 7:** Estimated incidence of advanced soft-tissue sarcoma in all of Germany in 2012.

Age (years)	German population	Estimated incidence rates of advanced STS^a^	Estimated number of cases of advanced STS
Male			
18–44	13,536,337	0.52	70
45–54	6,819,506	1.25	86
55–64	5,173,613	2.17	112
65–74	4,120,977	5.50	227
75+	3,029,828	8.57	260
Female			
18–44	13,199,959	0.46	60
45–54	6,688,226	1.27	85
55–64	5,359,480	3.47	186
65–74	4,628,276	3.85	178
75+	4,911,994	6.46	317
Total	67,468,196	2.31	1,581

^a^These are the cancer-specificmortality rates for patients with a diagnosis of STS; STS: soft-tissue sarcoma. Rates are per 100,000 inhabitants.

## Data Availability

The data on sarcoma cases used in this study were provided by the RKI/ZfKD under a data use agreement with RTI Health Solutions (RTI-HS), and so cannot be made freely available. Any interested third party can lodge their own application with the ZfKD.

## Supplementary Materials

Supplementary Materials Table S1: completeness of cancer registry data for each federal state, average 2003 through 2012. Table S2: soft-tissue sarcoma ICD-O-3 inclusion criteria codes. Table S3: ICD-O-3 morphology and topography codes mapped to broad groupings. Table S4: ICD-O-3 exclusion codes.
