# Supplementary material for: Epidemiology of Adult Soft-Tissue Sarcomas in Germany
Source: Sarcoma. 2018 Apr 4;2018:5671926. doi: 10.1155/2018/5671926 (PMC5904765; doi:10.1155/2018/5671926)
Supplement: Supplementary Materials — Table S1: completeness of cancer registry data for each federal state, average 2003 through 2012. Table S2: soft-tissue sarcoma ICD-O-3 inclusion criteria codes. Table S3: ICD-O-3 morphology and topography codes mapped to broad groupings. Table S4: ICD-O-3 exclusion codes. [file 5671926.f1.docx]

Supplementary Material

Contents

[Table S1. Completeness of cancer registry data for each federal state, average 2003 through 2012](#_Toc503444430)

[Table S2. Soft-tissue sarcoma ICD-O-3 inclusion criteria codes](#_Toc503444431)

[Table S3. ICD-O-3 morphology and topography codes mapped to broad groupings](#_Toc503444432)

[Table S4. ICD-O-3 exclusion codes](#_Toc503444433)

Table S1. Completeness of cancer registry data for each federal state, average 2003 through 2012

| Federal state | Start of registration | Percentage completeness | |
| --- | --- | --- | --- |
|  |  | All cancers combined | Sarcomas^a^ |
| Baden-Wuerttemberg | 2009 | < 80 | 90-95 |
| **Bavaria** | 2002 | > 95 | > 95 |
| Berlin | 1995 | 85-90 | < 80 |
| Brandenburg | 1995 | > 95 | 80-85 |
| **Bremen** | 1998 | > 95 | > 95 |
| **Hamburg** | 1991 | > 95 | > 95 |
| Hessen | 2007 | 80-85 | < 80 |
| **Lower Saxony** | 2003 | > 95 | 90-95 |
| Mecklenburg-Vorpommern | 1995 | 90-95 | < 80 |
| **North Rhine-Westphalia** | 2006 | > 95 | > 95 |
| **Rhineland-Palatinate** | 1999 | 90-95 | 90-95 |
| **Saarland** | 1970 | > 95 | > 95 |
| **Saxony** | 1995 | > 95 | > 95 |
| Saxony-Anhalt | 1995 | < 80 | < 80 |
| **Schleswig-Holstein** | 1998 | > 95 | > 95 |
| Thuringia | 1995 | 90-95 | < 80 |

^a^ Sarcomas are identified by ICD‑10 codes C40-C41 and C45-C49.

Note: If first year of registration was after 2003, then the period averaged is the first year of registration through 2012. Bolded states have at least 90% completeness of data for all cancers combined and for sarcomas.

Source: E-mail communication from Dr. K. Kraywinkel, German Centre for Cancer Registry, Robert Koch Institut, July 21, 2015.

| Table S2. Soft-tissue sarcoma ICD-O-3 inclusion criteria codes | |
| --- | --- |
| ICD-O-3 code | Notes |
| 8000 | if /3 and topography code begins with C49 |
| 8587 | if /3 |
| 8710 | if /3 |
| 8711 | if /3 |
| 8713 | if /3 |
| 8714 | if /3 |
| 8800 | if /3 and topography code does **not** begin with C40 or C41 (bone sites) |
| 8801 | if /3 |
| 8802 | if /3 |
| 8803 | if /3 |
| 8804 | if /3 |
| 8805 | if /3 |
| 8806 | if /3 |
| 8810 | if /3 |
| 8811 | if /3 |
| 8813 | if /3 |
| 8814 | if /3 |
| 8815 | if /3 |
| 8820 | if /3 |
| 8821 | if /3 |
| 8822 | if /3 |
| 8823 | if /3 |
| 8824 | if /3 |
| 8825 | if /3 |
| 8826 | if /3 |
| 8827 | if /3 |
| 8828 | if /3 |
| 8830 | if /3 |
| 8831 | if /3 |
| 8832 | if /3 |
| 8833 | if /3 |
| 8834 | if /3 |
| 8835 | if /3 |
| 8836 | if /3 |
| 8840 | if /3 |
| 8841 | if /3 |
| 8842 | if /3 |
| 8850 | if /3 |
| 8851 | if /3 |
| 8852 | if /3 |
| 8853 | if /3 |
| 8854 | if /3 |
| 8855 | if /3 |
| 8856 | if /3 |
| 8857 | if /3 |
| 8858 | if /3 |
| 8860 | if /3 |
| 8861 | if /3 |
| 8862 | if /3 |
| 8870 | if /3 |
| 8880 | if /3 |
| 8881 | if /3 |
| 8890 | if /3 |
| 8891 | if /3 |
| 8892 | if /3 |
| 8893 | if /3 |
| 8894 | if /3 |
| 8895 | if /3 |
| 8896 | if /3 |
| 8897 | if /3 |
| 8898 | if /3 |
| 8900 | if /3 |
| 8901 | if /3 |
| 8902 | if /3 |
| 8903 | if /3 |
| 8904 | if /3 |
| 8905 | if /3 |
| 8910 | if /3 |
| 8912 | if /3 |
| 8920 | if /3 |
| 8921 | if /3 |
| 8940 | if /3 |
| 8963 | if /3 |
| 8982 | if /3 |
| 8990 | if /3 |
| 9020 | if /3 |
| 9040 | if /3 |
| 9041 | if /3 |
| 9042 | if /3 |
| 9043 | if /3 |
| 9044 | if /3 |
| 9120 | if /3 |
| 9121 | if /3 |
| 9122 | if /3 |
| 9123 | if /3 |
| 9125 | if /3 |
| 9130 | if /3 |
| 9131 | if /3 |
| 9132 | if /3 |
| 9133 | if /3 |
| 9135 | if /3 |
| 9136 | if /3 |
| 9137 | if /3 |
| 9141 | if /3 |
| 9142 | if /3 |
| 9150 | if /3 |
| 9160 | if /3 |
| 9161 | if /3 |
| 9170 | if /3 |
| 9171 | if /3 |
| 9172 | if /3 |
| 9173 | if /3 |
| 9174 | if /3 |
| 9175 | if /3 |
| 9231 | if /3 |
| 9251 | if /3 |
| 9252 | if /3 |
| 9364 | if /3 |
| 9365 | if /3 |
| 9540 | if /3 |
| 9542 | if /3 |
| 9560 | if /3 |
| 9561 | if /3 |
| 9562 | if /3 |
| 9563 | if /3 |
| 9570 | if /3 |
| 9571 | if /3 |
| 9580 | if /3 |
| 9581 | if /3 |

| Table S3. ICD-O-3 morphology and topography codes mapped to broad groupings | |
| --- | --- |
| ICD-O-3 code | Group |
| 8850 | Liposarcoma |
| 8852 | Liposarcoma |
| 8854 | Liposarcoma |
| 8858 | Liposarcoma |
| 8851 | Liposarcoma |
| 8853 | Liposarcoma |
| 8855 | Liposarcoma |
| 8857 | Liposarcoma |
| 8815 | Fibroblastic/myofibroblastic |
| 8832 | Fibroblastic/myofibroblastic |
| 8810 | Fibroblastic/myofibroblastic |
| 8811 | Fibroblastic/myofibroblastic |
| 8814 | Fibroblastic/myofibroblastic |
| 8825 | Fibroblastic/myofibroblastic |
| 8840 | Fibroblastic/myofibroblastic |
| 8813 | Fibroblastic/myofibroblastic |
| 8821 | Fibroblastic/myofibroblastic |
| 8833 | Fibroblastic/myofibroblastic |
| 8000 | Malignant neoplasm arising in soft tissues, nos |
| 9540 | Nerve sheath |
| 9561 | Nerve sheath |
| 9571 | Nerve sheath |
| 9580 | Nerve sheath |
| 9560 | Nerve sheath |
| 9562 | Nerve sheath |
| 8711 | Pericytic |
| 9150 | Pericytic |
| 8800 | Sarcoma NOS |
| 8805 | Sarcoma NOS |
| 8901 | Skeletal muscle |
| 8910 | Skeletal muscle |
| 8912 | Skeletal muscle |
| 8920 | Skeletal muscle |
| 8900 | Skeletal muscle |
| 8902 | Skeletal muscle |
| 8890 | Leiomyosarcoma |
| 8891 | Leiomyosarcoma |
| 8895 | Leiomyosarcoma |
| 8896 | Leiomyosarcoma |
| 8830 | So-called fibrohistiocytic |
| 9252 | So-called fibrohistiocytic |
| 9251 | So-called fibrohistiocytic |
| 8804 | Tumors of uncertain differentiation |
| 8806 | Tumors of uncertain differentiation |
| 8940 | Tumors of uncertain differentiation |
| 8963 | Tumors of uncertain differentiation |
| 8982 | Tumors of uncertain differentiation |
| 8990 | Tumors of uncertain differentiation |
| 9040 | Tumors of uncertain differentiation |
| 9041 | Tumors of uncertain differentiation |
| 9043 | Tumors of uncertain differentiation |
| 9044 | Tumors of uncertain differentiation |
| 9231 | Tumors of uncertain differentiation |
| 9364 | Tumors of uncertain differentiation |
| 9581 | Tumors of uncertain differentiation |
| 9020 | Tumors of uncertain differentiation |
| 9042 | Tumors of uncertain differentiation |
| 9365 | Tumors of uncertain differentiation |
| 8801 | Undifferentiated/unclassified sarcomas |
| 8802 | Undifferentiated/unclassified sarcomas |
| 8803 | Undifferentiated/unclassified sarcomas |
| 9120 | Vascular tumors of soft tissue |
| 9133 | Vascular tumors of soft tissue |
| 8710 | Vascular tumors of soft tissue |
| 8860 | Vascular tumors of soft tissue |
| 8861 | Vascular tumors of soft tissue |
| 8894 | Vascular tumors of soft tissue |
| 9125 | Vascular tumors of soft tissue |
| 9130 | Vascular tumors of soft tissue |
| 9170 | Vascular tumors of soft tissue |
| C000 | Head or neck |
| C001 | Head or neck |
| C003 | Head or neck |
| C019 | Head or neck |
| C020 | Head or neck |
| C021 | Head or neck |
| C023 | Head or neck |
| C028 | Head or neck |
| C029 | Head or neck |
| C030 | Head or neck |
| C031 | Head or neck |
| C039 | Head or neck |
| C040 | Head or neck |
| C041 | Head or neck |
| C049 | Head or neck |
| C050 | Head or neck |
| C051 | Head or neck |
| C058 | Head or neck |
| C059 | Head or neck |
| C060 | Head or neck |
| C068 | Head or neck |
| C069 | Head or neck |
| C079 | Head or neck |
| C080 | Head or neck |
| C081 | Head or neck |
| C089 | Head or neck |
| C090 | Head or neck |
| C091 | Head or neck |
| C099 | Head or neck |
| C101 | Head or neck |
| C102 | Head or neck |
| C103 | Head or neck |
| C108 | Head or neck |
| C109 | Head or neck |
| C111 | Head or neck |
| C118 | Head or neck |
| C119 | Head or neck |
| C129 | Head or neck |
| C130 | Head or neck |
| C131 | Head or neck |
| C132 | Head or neck |
| C138 | Head or neck |
| C139 | Head or neck |
| C140 | Head or neck |
| C148 | Head or neck |
| C150 | Gastrointestinal |
| C151 | Gastrointestinal |
| C152 | Gastrointestinal |
| C153 | Gastrointestinal |
| C154 | Gastrointestinal |
| C155 | Gastrointestinal |
| C158 | Gastrointestinal |
| C159 | Gastrointestinal |
| C160 | Gastrointestinal |
| C161 | Gastrointestinal |
| C162 | Gastrointestinal |
| C163 | Gastrointestinal |
| C164 | Gastrointestinal |
| C165 | Gastrointestinal |
| C166 | Gastrointestinal |
| C168 | Gastrointestinal |
| C169 | Gastrointestinal |
| C170 | Gastrointestinal |
| C171 | Gastrointestinal |
| C172 | Gastrointestinal |
| C173 | Gastrointestinal |
| C178 | Gastrointestinal |
| C179 | Gastrointestinal |
| C180 | Gastrointestinal |
| C181 | Gastrointestinal |
| C182 | Gastrointestinal |
| C183 | Gastrointestinal |
| C184 | Gastrointestinal |
| C185 | Gastrointestinal |
| C186 | Gastrointestinal |
| C187 | Gastrointestinal |
| C188 | Gastrointestinal |
| C189 | Gastrointestinal |
| C199 | Gastrointestinal |
| C209 | Gastrointestinal |
| C210 | Gastrointestinal |
| C211 | Gastrointestinal |
| C218 | Gastrointestinal |
| C220 | Gastrointestinal |
| C221 | Gastrointestinal |
| C239 | Gastrointestinal |
| C240 | Gastrointestinal |
| C241 | Gastrointestinal |
| C249 | Gastrointestinal |
| C250 | Gastrointestinal |
| C251 | Gastrointestinal |
| C252 | Gastrointestinal |
| C258 | Gastrointestinal |
| C259 | Gastrointestinal |
| C260 | Gastrointestinal |
| C268 | Gastrointestinal |
| C269 | Gastrointestinal |
| C300 | Head or neck |
| C301 | Head or neck |
| C310 | Head or neck |
| C311 | Head or neck |
| C312 | Head or neck |
| C313 | Head or neck |
| C318 | Head or neck |
| C319 | Head or neck |
| C320 | Head or neck |
| C321 | Head or neck |
| C322 | Head or neck |
| C323 | Head or neck |
| C328 | Head or neck |
| C329 | Head or neck |
| C339 | Head or neck |
| C340 | Heart, mediastinum, lung, or pleura |
| C341 | Heart, mediastinum, lung, or pleura |
| C342 | Heart, mediastinum, lung, or pleura |
| C343 | Heart, mediastinum, lung, or pleura |
| C348 | Heart, mediastinum, lung, or pleura |
| C349 | Heart, mediastinum, lung, or pleura |
| C379 | Heart, mediastinum, lung, or pleura |
| C380 | Heart, mediastinum, lung, or pleura |
| C381 | Heart, mediastinum, lung, or pleura |
| C382 | Heart, mediastinum, lung, or pleura |
| C383 | Heart, mediastinum, lung, or pleura |
| C384 | Heart, mediastinum, lung, or pleura |
| C388 | Heart, mediastinum, lung, or pleura |
| C390 | Heart, mediastinum, lung, or pleura |
| C399 | Heart, mediastinum, lung, or pleura |
| C400 | Miscellaneous |
| C401 | Miscellaneous |
| C402 | Miscellaneous |
| C403 | Miscellaneous |
| C409 | Miscellaneous |
| C410 | Miscellaneous |
| C411 | Miscellaneous |
| C412 | Miscellaneous |
| C413 | Miscellaneous |
| C414 | Miscellaneous |
| C418 | Miscellaneous |
| C419 | Miscellaneous |
| C421 | Miscellaneous |
| C422 | Miscellaneous |
| C440 | Head or neck |
| C441 | Head or neck |
| C442 | Head or neck |
| C443 | Head or neck |
| C444 | Head or neck |
| C445 | Trunk |
| C446 | Upper extremity |
| C447 | Lower extremity |
| C448 | Miscellaneous |
| C449 | Miscellaneous |
| C470 | Head or neck |
| C471 | Upper extremity |
| C472 | Lower extremity |
| C473 | Trunk |
| C474 | Trunk |
| C475 | Pelvis (nonvisceral) |
| C476 | Trunk |
| C478 | Miscellaneous |
| C479 | Miscellaneous |
| C480 | Retroperitoneal |
| C481 | Retroperitoneal |
| C482 | Retroperitoneal |
| C488 | Retroperitoneal |
| C490 | Head or neck |
| C491 | Upper extremity |
| C492 | Lower extremity |
| C493 | Trunk |
| C494 | Trunk |
| C495 | Pelvis (nonvisceral) |
| C496 | Trunk |
| C498 | Miscellaneous |
| C499 | Miscellaneous |
| C500 | Breast |
| C501 | Breast |
| C502 | Breast |
| C503 | Breast |
| C504 | Breast |
| C505 | Breast |
| C506 | Breast |
| C508 | Breast |
| C509 | Breast |
| C510 | Gynecologic (other than uterus) |
| C511 | Gynecologic (other than uterus) |
| C512 | Gynecologic (other than uterus) |
| C518 | Gynecologic (other than uterus) |
| C519 | Gynecologic (other than uterus) |
| C529 | Gynecologic (other than uterus) |
| C530 | Uterus |
| C531 | Uterus |
| C538 | Uterus |
| C539 | Uterus |
| C540 | Uterus |
| C541 | Uterus |
| C542 | Uterus |
| C543 | Uterus |
| C548 | Uterus |
| C549 | Uterus |
| C559 | Uterus |
| C569 | Gynecologic (other than uterus) |
| C570 | Gynecologic (other than uterus) |
| C571 | Gynecologic (other than uterus) |
| C572 | Gynecologic (other than uterus) |
| C573 | Gynecologic (other than uterus) |
| C574 | Gynecologic (other than uterus) |
| C578 | Gynecologic (other than uterus) |
| C579 | Gynecologic (other than uterus) |
| C600 | Genitourinary |
| C601 | Genitourinary |
| C602 | Genitourinary |
| C608 | Genitourinary |
| C609 | Genitourinary |
| C619 | Genitourinary |
| C620 | Genitourinary |
| C621 | Genitourinary |
| C629 | Genitourinary |
| C630 | Genitourinary |
| C631 | Genitourinary |
| C632 | Genitourinary |
| C637 | Genitourinary |
| C638 | Genitourinary |
| C639 | Genitourinary |
| C649 | Genitourinary |
| C659 | Genitourinary |
| C669 | Genitourinary |
| C670 | Genitourinary |
| C671 | Genitourinary |
| C672 | Genitourinary |
| C673 | Genitourinary |
| C674 | Genitourinary |
| C675 | Genitourinary |
| C676 | Genitourinary |
| C677 | Genitourinary |
| C678 | Genitourinary |
| C679 | Genitourinary |
| C680 | Genitourinary |
| C688 | Genitourinary |
| C689 | Genitourinary |
| C690 | Head or neck |
| C695 | Head or neck |
| C696 | Head or neck |
| C700 | Miscellaneous |
| C701 | Miscellaneous |
| C709 | Miscellaneous |
| C710 | Miscellaneous |
| C711 | Miscellaneous |
| C712 | Miscellaneous |
| C713 | Miscellaneous |
| C714 | Miscellaneous |
| C715 | Miscellaneous |
| C716 | Miscellaneous |
| C717 | Miscellaneous |
| C718 | Miscellaneous |
| C719 | Miscellaneous |
| C720 | Miscellaneous |
| C721 | Miscellaneous |
| C722 | Miscellaneous |
| C723 | Miscellaneous |
| C724 | Miscellaneous |
| C725 | Miscellaneous |
| C728 | Miscellaneous |
| C729 | Miscellaneous |
| C739 | Miscellaneous |
| C740 | Miscellaneous |
| C741 | Miscellaneous |
| C749 | Miscellaneous |
| C751 | Miscellaneous |
| C754 | Miscellaneous |
| C755 | Miscellaneous |
| C759 | Miscellaneous |
| C760 | Head or neck |
| C761 | Miscellaneous |
| C762 | Miscellaneous |
| C763 | Trunk |
| C764 | Upper extremity |
| C765 | Lower extremity |
| C767 | Trunk |
| C768 | Miscellaneous |
| C809 | Unknown |

| Table S4. ICD-O-3 exclusion codes | |
| --- | --- |
| ICD-O-3 code | Description |
| 8936 | Gastrointestinal stromal sarcoma |
| 9140 | Kaposi sarcoma; multiple hemorrhagic sarcoma |
| 8812 | Bone sarcomas |
| 9180 | Bone sarcomas |
| 9181 | Bone sarcomas |
| 9182 | Bone sarcomas |
| 9183 | Bone sarcomas |
| 9184 | Bone sarcomas |
| 9185 | Bone sarcomas |
| 9186 | Bone sarcomas |
| 9187 | Bone sarcomas |
| 9191 | Bone sarcomas |
| 9192 | Bone sarcomas |
| 9193 | Bone sarcomas |
| 9194 | Bone sarcomas |
| 9195 | Bone sarcomas |
| 9200 | Bone sarcomas |
| 9210 | Bone sarcomas |
| 9220 | Bone sarcomas |
| 9221 | Bone sarcomas |
| 9230 | Bone sarcomas |
| 9240 | Bone sarcomas |
| 9241 | Bone sarcomas |
| 9242 | Bone sarcomas |
| 9243 | Bone sarcomas |
| 9250 | Bone sarcomas |
| 9260 | Bone sarcomas |
| 9261 | Bone sarcomas |
| 9270 | Bone sarcomas |
| 9290 | Bone sarcomas |
| 9310 | Bone sarcomas |
| 9330 | Bone sarcomas |
| 9341 | Bone sarcomas |
| 9342 | Bone sarcomas |
| 9370 | Bone sarcomas |
| 9371 | Bone sarcomas |
| 9372 | Bone sarcomas |
| 9373 | Bone sarcomas |
